# Differential requirement for satellite cells during overload-induced muscle hypertrophy in growing versus mature mice

**DOI:** 10.1186/s13395-017-0132-z

**Published:** 2017-07-10

**Authors:** Kevin A. Murach, Sarah H. White, Yuan Wen, Angel Ho, Esther E. Dupont-Versteegden, John J. McCarthy, Charlotte A. Peterson

**Affiliations:** 1 Department of Rehabilitation Sciences, College of Health Sciences, 900 South Limestone, Lexington, KY 40536 USA; 2 Department of Physiology, College of Medicine, 800 Rose Street, Lexington, KY 40536 USA; 3 The Center for Muscle Biology, University of Kentucky, 900 South Limestone, Lexington, KY 40536 USA; 4 Department of Animal Science, Texas A&M University, College Station, TX 77843 USA

**Keywords:** Pax7, Synergist ablation, Regeneration, Fiber splitting, Development, Muscle hypertrophy

## Abstract

**Background:**

Pax7+ satellite cells are required for skeletal muscle fiber growth during post-natal development in mice. Satellite cell-mediated myonuclear accretion also appears to persist into early adulthood. Given the important role of satellite cells during muscle development, we hypothesized that the necessity of satellite cells for adaptation to an imposed hypertrophic stimulus depends on maturational age.

**Methods:**

Pax7^CreER^-R26R^DTA^ mice were treated for 5 days with vehicle (satellite cell-replete, SC+) or tamoxifen (satellite cell-depleted, SC-) at 2 months (young) and 4 months (mature) of age. Following a 2-week washout, mice were subjected to sham surgery or 10 day synergist ablation overload of the plantaris (*n* = 6–9 per group). The surgical approach minimized regeneration, de novo fiber formation, and fiber splitting while promoting muscle fiber growth. Satellite cell density (Pax7+ cells/fiber), embryonic myosin heavy chain expression (eMyHC), and muscle fiber cross sectional area (CSA) were evaluated via immunohistochemistry. Myonuclei (myonuclei/100 mm) were counted on isolated single muscle fibers.

**Results:**

Tamoxifen treatment depleted satellite cells by ≥90% and prevented myonuclear accretion with overload in young and mature mice (*p* < 0.05). Satellite cells did not recover in SC- mice after overload. Average muscle fiber CSA increased ~20% in young SC+ (*p* = 0.07), mature SC+ (*p* < 0.05), and mature SC- mice (*p* < 0.05). In contrast, muscle fiber hypertrophy was prevented in young SC- mice. Muscle fiber number increased only in mature mice after overload (*p* < 0.05), and eMyHC expression was variable, specifically in mature SC+ mice.

**Conclusions:**

Reliance on satellite cells for overload-induced hypertrophy is dependent on maturational age, and global responses to overload differ in young versus mature mice.

**Electronic supplementary material:**

The online version of this article (doi:10.1186/s13395-017-0132-z) contains supplementary material, which is available to authorized users.

## Background

Satellite cells (Pax7+) comprise the predominant skeletal muscle stem cell population. Located between the muscle fiber plasma membrane and the basal lamina [1, 2], these cells are activated in response to muscle injury and are indispensable for muscle fiber regeneration [3–6]. Most evidence supports the conclusion that myonuclei are post-mitotic [7–11], so another role for satellite cells is to fuse into muscle fibers for the purpose of myonuclear addition and/or replacement. During muscle fiber hypertrophy, satellite cell-mediated myonuclear accretion stabilizes the “myonuclear domain,” a hypothetically fixed area of the cytoplasm that each myonucleus transcriptionally governs [12–16]. The theorized importance of maintaining the myonuclear domain underpins the argument that satellite cells are necessary for muscle fiber hypertrophy.

Contrary to the myonuclear domain theory of muscle growth, numerous investigations report robust muscle fiber hypertrophy in the absence of satellite cell-mediated myonuclear accretion [4, 17–26]. Utilizing the Pax7^CreER^-R26R^DTA^ tamoxifen-inducible mouse model, our laboratory showed similar hypertrophic responses in vehicle-treated (satellite cell-replete, SC+) versus tamoxifen-treated (satellite cell-depleted, SC-) adult mice after 2 weeks of mechanical overload of the plantaris [4, 27]. We subsequently found that resident myonuclei in adult SC- mice can robustly up-regulate transcriptional output to meet biosynthetic demands during the early phase of hypertrophy [26]. Following an extended overload period (8 weeks) in SC- mice, average muscle fiber size increased >25%, but pronounced extracellular matrix (ECM) deposition appeared to attenuate muscle fiber hypertrophy [25]. Prolonged absence of satellite cells causes aberrant ECM accumulation under various conditions [5, 24, 25, 28, 29], as activated satellite cells directly communicate with fibroblasts to coordinate ECM dynamics during adaptation [24]. However, the presence of satellite cells during the first week of plantaris overload was sufficient to ensure proper ECM remodeling; deletion of satellite cells after the first week resulted in uncompromised muscle fiber hypertrophy with minimal myonuclear accretion by 8 weeks [24]. Collectively, these data indicate that striking muscle fiber hypertrophy (and consequently myonuclear domain expansion) occurs in the absence of significant satellite cell-mediated myonuclear accretion.

All investigations utilizing the Pax7^CreER^-R26R^DTA^ model from our laboratory have involved mature mice ≥4 months of age. Although some evidence indicates that satellite cell fusion into muscle fibers is complete by post-natal day 21 [30], others report that satellite cells contribute to muscle fibers beyond 2 months of age [31, 32], suggesting a maturational reliance on satellite cells into early adulthood that may have consequences for adaptation. The primary purpose of this investigation was to rigorously test the requirement for satellite cell-mediated myonuclear accretion during overload in young mice. We hypothesized that young mice would have a more stringent requirement for satellite cells during adaptation to overload than mature mice.

## Methods

### Mice

All animal procedures were carried out in accordance with institutional guidelines for the care and use of laboratory animals, as approved by the Institutional Animal Care and Use Committee of the University of Kentucky. Mice were housed in a temperature- and humidity-controlled room with a 14:10-light-dark cycle. Food and water were provided ad libitum. The Pax7^CreER/+^-R26R^DTA/+^ strain, designated Pax7-DTA from here onward, was generated by crossing the Pax7^CreER/CreER^ mouse strain from the Kardon laboratory [5] with the Rosa26^DTA/DTA^ mouse strain. Upon tamoxifen administration, *Cre*-mediated recombination drives expression of diphtheria toxin A chain (DTA), thereby killing satellite cells that express the *Pax7* gene. As a control, mice from the parental strain, Pax7^CreER/CreER^, designated Pax7-CreER, were also utilized (*n* = 3 per group). Male C57BL6 mice >4 months of age were obtained from the Jackson Laboratory (Bar Harbor, ME, USA).

### Conditional ablation of satellite cells

**Fig. 1:**
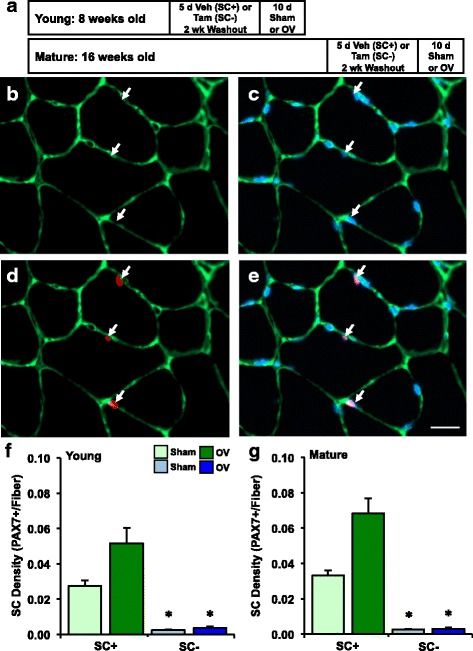
Conditional depletion of satellite cells (Pax7+) in young and mature mice before synergist ablation surgery to overload the plantaris muscle for 10 days (OV). **a** Study design schematic demonstrating the duration of vehicle (satellite cell-replete, SC+) and tamoxifen (satellite cell-depleted, SC-) treatment, washout, and sham or OV surgery in young and mature mice. **b**–**e** Immunohistochemistry (IHC) representative images visualizing **b** laminin (*green*), **c** myonuclei (*blue*), **d** Pax7 (*red*), and **e** merged fields, where satellite cells are identified as Pax7+/DAPI+. *White arrows* denote the same satellite cell in each image; scale bar=20 μm. **f** Satellite cell density in young sham (*n* = 7 SC+, *n* = 6 SC-) and OV (*n* = 8 SC+, *n* = 6 SC-) mice. **g** Satellite cell density in mature sham (*n* = 9 SC+, *n* = 7 SC-) and OV (*n* = 6 SC+, *n* = 7 SC-) mice. *p < 0.05 compared to analogous SC+ group

A study design schematic is found in Fig. 1a. Young (8-week-old) and mature (16-week-old) male mice were treated with vehicle (15% ethanol in sunflower seed oil) or tamoxifen (2 mg/d) for five consecutive days. Following a 2-week washout period, mice were randomly divided into sham surgery or bilateral synergist ablation surgery groups. Mice were overloaded for 10 days and then sacrificed in order to harvest the plantaris muscles. Pax7-CreER mice were also injected with vehicle or tamoxifen at 8 weeks of age, allowed a 2-week washout, then subjected to sham surgery or synergist ablation surgery to determine the effects of tamoxifen treatment on the hypertrophic response, independent from satellite cell depletion. Results of this experiment in mature mice were published previously by our laboratory [25].

### Synergist ablation surgery

To overload the plantaris of Pax7-DTA mice, we employed a modified version of the bilateral synergist ablation surgical approach. Traditionally, this surgery involves removal of a significant portion of the gastrocnemius and soleus muscles, thereby overloading the synergist plantaris muscle [26]. To minimize regeneration, de novo fiber formation, and increased fiber number that is often associated with the aforementioned approach [4, 33], we modified the surgery. Mice were anesthetized using isoflurane under sterile conditions, and a longitudinal incision on the dorsal aspect of the hindlimb exposed the Achilles tendon. Carefully probing the plantaris tendon away from the Achilles, the soleus-gastrocnemius complex was isolated, and the tendon, myotendinous junction, and a small portion of gastrocnemius and soleus muscles (~5 mg) were excised without disturbing the innervation or blood supply to the plantaris. The sham surgery consisted of all the aforementioned steps except removal of the tendon and muscle. Following 10 days of overload or normal ambulation, mice (*n* = 6–9 per group for both young and mature) were euthanized, plantaris muscles were harvested and weighed, and appropriately processed for immunohistochemical or single muscle fiber analyses.

To gain further insight into the hypertrophic responses to a more severe overload stimulus, we re-analyzed the effects of the traditional 14-day bilateral synergist ablation approach used previously by our laboratory in greater detail [4]. We also conducted a 14-day “synergist tenotomy” surgical overload approach in C57BL6 mice. This surgery involved careful removal of the gastrocnemius tendon and a small portion of medial and lateral gastrocnemius muscles, leaving the soleus and plantaris intact. Since the soleus provides support to the plantaris using this approach, a longer overload period without significant muscle damage is possible. Plantaris muscles were harvested and processed as described above.

### Immunohistochemistry and single fiber analysis

For immunohistochemistry (IHC) analyses, the plantaris muscle was covered in Tissue-Tek optimal cutting temperature compound (Sakura Finetek, Torrance, CA, USA) and pinned at resting length to a cork covered in aluminum foil. Muscles were frozen in liquid nitrogen-cooled isopentane and stored at −80 °C. At the time of sectioning, muscles were trimmed to mid-belly using a razor blade and oriented upright in tissue Tissue-Tek, which was then frozen using freeze spray in order to secure the muscle sample in place. Mid-belly plantaris muscle sections were cut on a cryostat at −23 °C. Frozen muscle sections (7 μm) were air-dried for ≥2 h and stored at −20 °C. For Pax7/Laminin/DAPI IHC, muscles were fixed in 4% paraformaldehyde for 7 min, incubated with rabbit anti-laminin IgG antibody overnight (1:100, L9393, Sigma-Aldrich, St. Louis, MO, USA), then incubated with Alexa Fluor 488 goat anti-rabbit IgG (1:250, A11034, Invitrogen, Carlsbad, CA, USA) for 1 h. Following epitope retrieval in sodium citrate (10 mM, pH 6.5) for 20 min at 92 °C, endogenous peroxidases were blocked for 7 min with 3% hydrogen peroxide in phosphate-buffered saline (PBS), followed by 1 h with 1% Tyramide Signal Amplification (TSA) blocking reagent (TSA kit, T20935, Invitrogen) supplemented with Mouse-on-Mouse (MoM) IgG blocking reagent (Vector Laboratories, Burlingame, CA, USA). Sections were washed in PBS and incubated overnight with mouse anti-Pax7 IgG1 antibody (1:100, Developmental Studies Hybridoma Bank (DHSB), Iowa City, IA, USA) diluted in 1% TSA blocking reagent. The next day, sections were washed with PBS, incubated for 70 min in goat anti-mouse IgG1 biotinylated secondary antibody (1:1000, 115-065-205, Jackson ImmunoResearch, West Grove, PA, USA), washed in PBS, incubate for 1 h in streptavidin-horseradish peroxidase (1:500, S-911, Invitrogen) diluted in PBS, washed again in PBS, then incubated for 15 min in TSA Alexa Fluor 594 (1:100, TSA kit, Invitrogen) in the supplied amplification diluents. Sections were stained with DAPI (1:10,000 in PBS, D35471, Invitrogen) for 5 min and mounted with VectaShield fluorescent mounting media (Vector).

To quantify embryonic myosin heavy chain (eMyHC) and fiber cross-sectional area (CSA), sections were incubated for 1 h with MoM, then incubated overnight with MyH3 anti-eMyHC IgG1 (Neat, F1.652, DHSB) and anti-laminin (1:100, Sigma Aldrich). The following day, sections were washed and incubated with goat anti-mouse Alexa Fluor 488 IgG1 (1:250, A-21121, Invitrogen) and Alexa Fluor 647 donkey anti-rabbit IgG (1:100, A31573, Invitrogen) for 1 h, stained with DAPI (Invitrogen), then mounted with VectaShield (Vector). In C57BL6 mice, dystrophin IHC was used to visualize fiber borders. Frozen sections were blocked with MoM (Vector), incubated overnight in primary antibody against dystrophin (1:100, VPD505, Vector), incubated with IgG1 Alexa Fluor 488 (1:250, A-21121, Invitrogen), and mounted with Vectashield (Vector).

Myonuclear counts were conducted on isolated single muscle fibers, as previously described by our laboratory and others [4, 34]. Briefly, plantaris muscles of sham and overloaded mice were fixed in situ at resting length in 4% paraformaldehyde for 48 h. Fixed whole muscles were removed from the hindlimb, manually dissected apart, then dissociated in 40% sodium hydroxide at room temperature. Isolated fibers were then stained with DAPI and carefully pipetted on to glass slides and covered using Vectashield. Thirty fibers per mouse were used for myonuclear density analysis.

### Immunohistochemistry and single muscle fiber myonuclear quantification

For IHC, images were captured at ×20 magnification at room temperature using a Zeiss upright fluorescent microscope (Zeiss AxioImager M1 Oberkochen, Germany). Whole muscle sections were obtained using the mosaic function in Zeiss Zen 2.3 imaging software. Capturing the entire muscle cross section is important for quantifying average muscle fiber cross CSA; counting all fibers eliminates the possibility of bias due to the regional nature of the plantaris and accounts for the potential effects of increased fiber number via de novo muscle fiber formation and/or fiber splitting. Muscle fiber cross-sectional area and eMyHC proportion were analyzed using custom software, developed by the University of Kentucky Center for Muscle Biology. This robust and highly sensitive muscle analysis software has been validated against manual human counts and is both accurate and reliable. Centrally nucleated muscle fibers were quantified manually. For myonuclear analysis, single muscle fibers were imaged at ×20 magnification using the Z-stack function within the Zen software. To determine satellite cell density (Pax7+ cells/fiber), satellite cells (Pax7+/DAPI+) were counted manually using tools in the Zen software. Satellite cell counts were normalized to fiber number, delineated by laminin+ boundaries. All manual counting was performed by a blinded, trained technician.

### Statistical analysis

Two-way ANOVAs within each group (young and mature) utilizing original or log-transformed data were used for analysis. When an interaction was found, post hoc pairwise comparisons were made using the Fisher’s procedure, and Tukey’s pairwise comparisons were used otherwise. Sham mice did not express eMyHC in any group; independent *t* tests were used to compare eMyHC expression after overload in young and mature mice. Significance was set at *p* ≤ 0.05, and trends were reported if *p* ≤ 0.10. Data are presented as mean ± standard error. All statistical analyses were performed in JMP statistical software (SAS, Cary, NC).

## Results

### Satellite cells are effectively depleted with tamoxifen administration and do not recover after overload

Following tamoxifen treatment in Pax7-DTA mice, we routinely observed satellite cell depletion ≥90% in young and mature mice (*p* < 0.0001), as determined by Pax7 IHC (Figs. 1b–e). Mice <90% depleted were not included in the analysis. In SC- mice, the few remaining satellite cells did not proliferate and replenish the satellite pool after overload. Due to variability in SC+ mice, the apparent increase in satellite cell density (Pax7+/fiber) did not reach significance with overload relative to sham mice (+61% in young, +69% in mature, Figs. 1f–g). However, when the aberrant low responder in each overload group is removed, the increase in satellite cell density approached significance in both young (+69%, *p* = 0.06) and mature (+79%, *p* = 0.07) SC+ mice. We previously reported that removing a larger portion of the gastrocnemius and soleus to elicit greater overload of the plantaris (see “Methods”) resulted in a 360% increase in satellite cell density in mature SC+ mice [4]. The less pronounced satellite cell proliferation highlights that the approach used here is a comparatively less extreme and more translatable model to human muscle biology. The magnitude of satellite cell proliferation in SC+ mice here is similar to what is observed in human models of exercise-induced hypertrophy [35–40].

### Myonuclear accretion after 10 days of overload is prevented in satellite cell-depleted mice

**Fig. 2:**
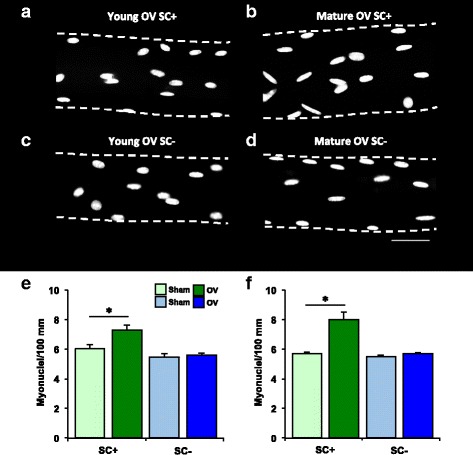
Satellite cell-mediated myonuclear accretion is prevented in satellite cell-depleted (SC-) but not satellite cell-replete (SC+) mice after synergist ablation overload of the plantaris for 10 days (OV). **a**–**d** Representative image of myonuclei per fiber length after OV in **a** young SC+, **b** mature SC+, **c** young SC-, **d** and mature SC- mice; scale bar=20 μm. **e** Myonuclei per 100 mm in young sham (*n* = 6 SC+, *n* = 5 SC-) and OV (*n* = 6 SC+, *n* = 6 SC-) mice. **f** Myonuclei per 100 mm in mature sham (*n* = 9 SC+, *n* = 7 SC-) and OV (*n* = 6 SC+, *n* = 7 SC-) mice. *p < 0.05 compared to respective sham

Myonuclei on isolated single muscle fibers were counted to quantify satellite cell-mediated myonuclear accretion, as previously described [4, 34]. Representative images of myonuclei per fiber for young and mature mice are shown in Figs. 2a–d. With overload in young and mature SC+ mice, myonuclei/100 mm increased 21% (*p* = 0.009) and 31% (*p* < 0.0001), respectively, relative to SC+ shams (Figs. 2e–f). Myonuclear accretion with overload was essentially eliminated following satellite cell depletion in young and mature mice, consistent with our previous reports in mature mice [4, 24, 25, 41]. Although there appeared to be fewer myonuclei in young sham SC- mice relative to young sham SC+ mice, the difference was not significant.

### Changes in eMyHC expression and fiber number after overload are different between young and mature mice

**Fig. 3:**
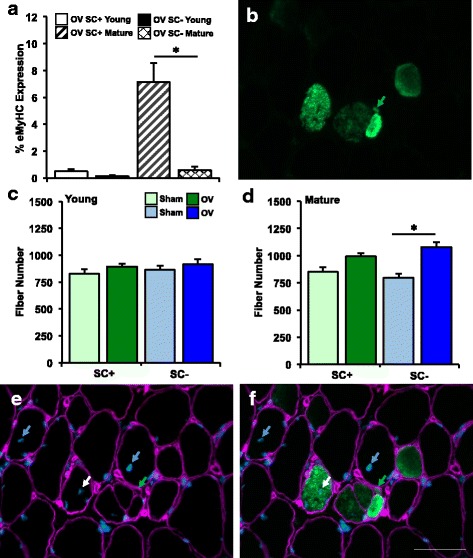
Changes in embryonic myosin heavy chain expression (eMyHC) and muscle fiber number per whole cross section in young versus mature satellite cell-replete (SC+) and -depleted (SC-) mice after synergist ablation overload of the plantaris for 10 days (OV). **a** eMyHC expression after OV in young (*n* = 8 SC+, *n* = 6 SC-) and mature (*n* = 7 SC+, *n* = 7 SC-) mice, *p < 0.05 SC+ versus SC- OV within each age group. **b** IHC representative image visualizing eMyHC fibers of differing size in a mature SC+ mouse; *green arrow* points to a small-caliber eMyHC+ muscle fiber <300 μm^2^. **c** Muscle fiber number in young sham (*n* = 7 SC+, *n* = 6 SC-) and OV (*n* = 8 SC+, *n* = 6 SC-) mice. **d** Muscle fiber number in mature sham (*n* = 9 SC+, *n* = 7 SC-) and OV (*n* = 6 SC+, *n* = 7 SC-) mice. *p < 0.05 compared to respective sham. **e**–**f** Representative image showing central nuclei (*white arrows*) in eMyHC+ (*green*) and eMyHC- (unstained) muscle fibers (*pink borders*). Scale bar=50 μm

To quantify the regenerative response to overload, eMyHC expression, muscle fiber number, and centrally nucleated muscle fibers were counted in young and mature mice (Fig. 3). In all sham-operated mice, eMyHC+ fibers were very rare (<0.1%), so these animals were not included in further analyses. Analysis of eMyHC expression in response to overload is shown in Fig. 3a, and a representative image of eMyHC expression in a mature overloaded SC+ mouse is shown in Fig. 3b. Consistent with our previous report in mature mice [4], eMyHC+ fibers were on average <1% (range, 0.0–1.7%) in all overloaded SC- mice regardless of age. In SC+ mice after overload, eMyHC-expressing fibers remained low to undetectable in young mice (range, 0.1–1.4%). In mature SC+ mice following overload, eMyHC+ fibers were more abundant (range, 0.2–18.4%) suggesting that, although variable, mature mice are more susceptible to a regenerative response in this surgical model. This is also consistent with the observation that muscle fiber number did not increase regardless of satellite cell content or overload in young mice (Fig. 3c), whereas muscle fiber number after overload increased in mature mice (Fig. 3d). However, because the fiber number increase was not significantly affected by the frequency of eMyHC+ fibers in mature mice, we analyzed the number of centrally nucleated muscle fibers as an additional marker of muscle fiber repair/regeneration. Figures 3e–f show that central nuclei (CN) occur in both eMyHC+ and eMyHC- muscle fibers in mature mice, indicating that these occurrences can be uncoupled (white and blue arrows, Figs. 3e–f). Therefore, CN+/eMyHC+ and CN+/eMyHC- muscle fibers were quantified separately. CN+/eMyHC+ fibers that are small in caliber (<300 μm^2^) and intensely stained for eMyHC are likely de novo formed fibers derived from satellite cells (green arrows, Figs. 3b, e–f). However, some large fibers also expressed high levels of eMyHC and contained a central nucleus (white arrows, Figs. 3e–f), which may represent rapid growth of a de novo formed fiber, or satellite cell-mediated fiber repair. In mature mice after overload, on average, 3% of all fibers were CN+/eMyHC+ in SC+ mice; only two SC- mice had any of these fibers, and they were very rare (<1%). The frequency of CN+/eMyHC- fibers with overload was comparable between SC+ and SC- mature mice, averaging 1–3% of the fibers, suggesting that CN+/eMyHC- fibers are not dependent on satellite cells.

To further explore the possibility that central nuclei can occur independent of a satellite cell-mediated regenerative event, we re-evaluated 5-bromo-2′-deoxyuridine (BrdU) incorporation in central nuclei from a previous overload investigation conducted by our laboratory [4]. Both BrdU+ and BrdU- central nuclei are apparent following overload in mature mice (Additional file 1), suggesting that some, but not all, central myonuclei may be derived from satellite cells that proliferated prior to fusion.

### Overload-induced muscle fiber hypertrophy is attenuated in young, but not mature SC- mice

**Fig. 4:**
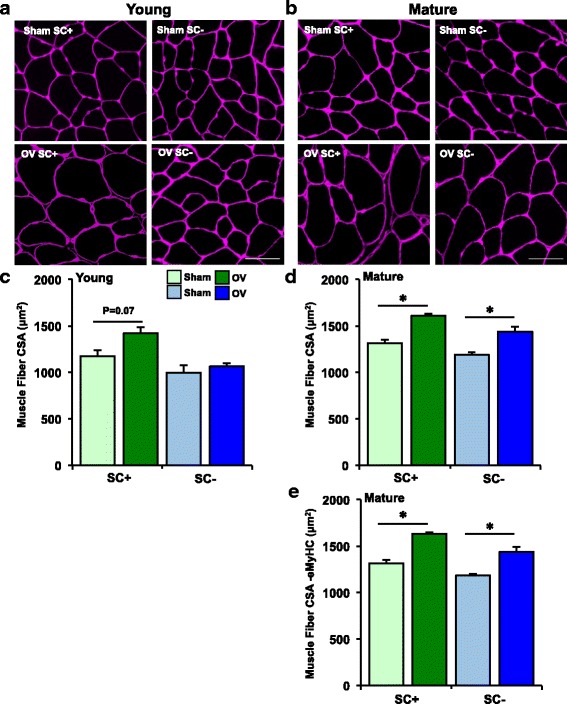
Average muscle fiber cross-sectional area (CSA) increases in young and mature satellite cell-replete (SC+) and mature satellite cell-depleted mice (SC-), but not young SC- mice after synergist ablation overload of the plantaris for 10 days (OV). IHC representative images visualizing muscle fiber borders (*pink*) in **a** young sham (SC+, SC-) and OV (SC+, SC-) mice, and **b** mature sham (SC+, SC-) and OV (SC+, SC-) mice; scale bar=20 μm. **c** Average muscle fiber CSA in young sham (*n* = 7 SC+, *n* = 6 SC-) and OV (*n* = 8 SC+, *n* = 6 SC-) mice, excluding fibers <300 μm^2^. **d** Average muscle fiber CSA in mature sham (*n* = 9 SC+, *n* = 7 SC-) and OV (*n* = 6 SC+, *n* = 7 SC-) mice, excluding fibers <300 μm^2^, but including eMyHC+ fibers. **e** Average muscle fiber CSA in mature mice, excluding fibers <300 μm^2^, as well as eMyHC+ fibers. *p < 0.05 compared to respective sham

Average muscle fiber size in response to overload in young and mature mice is shown in Fig. 4, and muscle fiber size frequency distributions are found in Additional file 2. Fibers with a cross-sectional area (CSA) <300 μm^2^ were omitted from these analyses in an attempt exclude de novo fibers (see green arrows, Fig. 3e–f), whereas eMyHC fibers >300 μm^2^ were included. Larger muscle fiber CSA after overload in young SC+ mice approached significance (19%, *p* = 0.07), but no increase was apparent in young SC- mice. The hypertrophic response to overload in young parental Pax7-CreER mice did not differ following vehicle or tamoxifen treatment (Additional file 3), indicating that the lack of hypertrophy in young SC- mice is attributable to satellite cell depletion and not Cre/Tamoxifen toxicity. Average muscle fiber CSA increased 20% in both SC+ and SC- mature mice (*p* < 0.05). When all eMyHC fibers were excluded in mature mice (very few eMyHC+ fibers were present in young mice), hypertrophy of SC+ muscle fibers was slightly greater than when eMyHC+ fibers were included (22%, *p* < 0.0001 relative to sham). Body weights, absolute plantaris weights, and plantaris weights normalized to body weights for young and mature groups are presented in Additional file 4. Body weight did not change with satellite cell depletion or overload in young or mature mice. Absolute plantaris weight increased in young SC+, mature SC+, and mature SC- mice with overload (*p* < 0.05), but not young SC- mice. Plantaris weight normalized to body weight increased in all overloaded young and mature mice, regardless of satellite cells (*p* < 0.05).

### Alternative adaptations to overload in mature mice

Increased fiber number and the appearance of CN+/eMyHC- fibers in mature mice suggested that muscle growth may not be entirely reflected by increased fiber CSA after overload. To explore this possibility, a more prolonged overload approach (14 day synergist tenotomy in C57BL6 mice, see “Methods” section) was employed in an effort to increase the frequency of these events without inducing significant regeneration. In eMyHC- fibers (<2% of fibers on average), we sometimes observed centrally nucleated muscle fibers concomitant with overt signs of muscle fiber splitting on histological cross sections (Additional file 5A–E), as well as longitudinal fiber splitting in isolated single muscle fibers (Additional file 5F). Fiber splitting is a characteristic of extreme overload, and the process seems coupled to muscle fiber central nucleation.

## Discussion

This investigation reports significantly different growth responses in SC+ and SC- mice across a relatively small maturational time frame: 11 weeks compared to 19 weeks of age. With mechanical overload, satellite cell depletion in young mice (at 2 months of age) prevents hypertrophic growth measured by fiber CSA, whereas skeletal muscle fibers in mature adult mice (>4 months of age) grow following satellite cell depletion and the absence of myonuclear accretion. Young mice also display different responses to overload relative to mature mice; eMyHC-expressing fibers do not increase in abundance, and fiber number does not increase, regardless of the presence of satellite cells in young mice. Our laboratory previously reported that myonuclei in adult mice have a robust transcriptional reserve capacity to support muscle fiber hypertrophy in the absence of satellite cell-mediated myonuclear accretion [26]. Future investigations will determine if myonuclei of young mice lack this reserve capacity, or whether immature myonuclei are transcriptionally unresponsive to increased tension. The requirements for satellite cells during adaptation seem to change along a dynamic continuum throughout the lifespan, and the age at which muscle overload experiments are conducted can profoundly affect outcomes.

Abnormally small muscle fibers with few myonuclei are reported in mice that survive germline deletion of Pax7 [42–44]. These findings underscore the importance of Pax7+ satellite cells for myonuclear accretion and growth during post-natal skeletal muscle development. The extensor digitorum longus (EDL), a non-weight-bearing muscle of the mouse hindlimb, reportedly reaches an asymptote in myonuclear accretion by post-natal day 21 [30]. On the other hand, the Gundersen laboratory reported significant myonuclear accretion beyond 2 months of age in the soleus (weight bearing) and EDL of mice when monitoring myonuclear number throughout the lifespan [31]. The Olwin laboratory also reported that satellite cell contribution to muscle fibers does not stabilize until ~3 months of age [32]. Collectively, these data suggest that mice in this age range are within a critical period of skeletal muscle development/maturation, potentially tied to long bone growth [45], that is dependent on satellite cells. We hypothesize that the satellite cell-dependency during this maturational period is associated with a stringent requirement for satellite cells to adapt to an imposed hypertrophic stimulus. Our results suggest that a transition occurs between 2.5 and 4 months, so that mice >4 months of age can grow in the absence of satellite cells by expanding the myonuclear domain. Two recent studies from other laboratories show attenuated muscle fiber hypertrophy when satellite cell-mediated myonuclear accretion was prevented in mice that were ~3 [46] or 3–4 months of age [47]. It is possible that mice in these investigations were still within this critical window of satellite cell dependence, but usage of different mouse strains [46], plantaris overload technique [46, 47], and tamoxifen treatment strategy [46] may also contribute to the lack of growth in the aforementioned studies.

Robust muscle fiber hypertrophy (>25%) occurs with minimal or non-existent satellite cell-mediated myonuclear accretion after 8 weeks of synergist ablation in mature mice (>4 months of age) [24, 25]. We now show average muscle fiber hypertrophy of ~20% in mature mice without satellite cells utilizing a short term (10 days) modified synergist ablation approach. Despite a marked shift to larger muscle fibers, a previous investigation from our laboratory showed ~10% increases in average muscle fiber CSA in mature SC+ and SC- mice after 14 days of synergist ablation [4]. We attributed the relatively modest increase in average muscle fiber CSA to a preponderance of newly formed small-caliber muscle fibers, which were revealed by analyzing all fibers in the entire plantaris cross section [4, 27]. Since muscle fiber number increased in both SC+ and SC- mice in that investigation, these new fibers were not exclusively satellite cell-derived. When revisiting the 14-day overload time point, we observed obvious signs of muscle fiber splitting. Fiber splitting during robust overload may therefore explain the relatively modest increase in average fiber CSA in our previous 14-day study [4].

Fiber splitting is well documented in response to various overload strategies in animals [48–57]. However, it is generally not well understood and typically disregarded as a hypertrophy mechanism in post-natal skeletal muscle [53, 58]. On muscle cross sections after overload, fiber splitting can be readily identified by faint or partial muscle fiber borders concomitant with centrally located myonuclei, uncoupled from regeneration (i.e., eMyHC expression) (Additional file 5). Consistent with earlier reports, central myonuclei appear to be a prominent feature of muscle fiber splitting [50, 52, 57, 59]. Furthermore, central myonuclei that arise during extreme overload in mice may or may not be BrdU+, suggesting that these nuclei are not necessarily derived from satellite cells (Additional file 1). Interestingly, extreme muscle fiber hypertrophy in bodybuilders and powerlifters produces fiber splitting morphology similar to what we observe with overload in mice, and the phenotype is exaggerated by enhanced hypertrophy and/or loading facilitated by anabolic steroid usage [59–63]. Thus, fiber splitting may constitute a non-pathological response to severe overload, perhaps to maintain oxygen diffusion distances when fibers grow too large [64, 65].

We did not observe overt signs of muscle fiber splitting after 10 days in our modified, minimally invasive overload approach utilized here. CN+/eMyHC- fibers did increase in mature SC+ and SC- mice after overload, but the overall prevalence was very low. While fiber splitting cannot be definitively ruled out, increased fiber number detected on muscle cross sections after overload in mature mice may be due to a change in muscle geometry, specifically pennation angle, which is known to occur during muscle growth [33, 66, 67]. The extent to which this accounts for the apparent change in fiber number in plantaris muscle cross sections in mature mice after mechanical overload remains to be determined. With the exception of a small number of eMyHC+ fibers <300 μm^2^ only in SC+ mice, which did not contribute significantly to overall muscle size, increased fiber CSA and increased fiber number were comparable in mature SC+ and SC- mice in response to overload. The shape of the fiber size frequency distribution curve also did not differ between mature SC+ and SC- mice after overload, suggesting a homogenous hypertrophic response (see Additional file 2). Our data collectively indicate that the nature and duration of overload surgery, as well as taking into account the total number of fibers in the muscle, are important considerations for accurate characterization of the hypertrophic response.

The lack of muscle fiber number increase in young SC+ mice, combined with the almost complete absence of eMyHC expression, suggest that maturational age affects the fundamental response to overload. Some potential explanations for these unique responses in young mice are (1) absolute body mass is not yet great enough to elicit the same overload response that mature mice experience during normal ambulation, (2) young mice are less susceptible to muscle damage and remodeling due to differences in the extracellular milieu, (3) young mice have an accelerated regenerative response, and/or (4) there are differences in activity level (and therefore, loading frequency and/or intensity) between young and mature mice. Regardless of the explanation, divergent hypertrophic responses depending on age must also be considered when conducting overload investigations in mice.

**Fig. 5:**
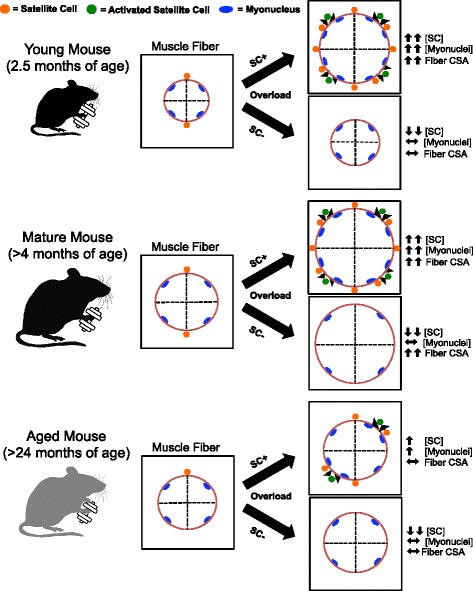
Dynamic continuum of satellite cell (SC) participation in overload-induced muscle fiber hypertrophy throughout the lifespan. Satellite cells are required for hypertrophic growth in young adult mice (2.5 months of age), are not required for hypertrophic growth in mature adult mice (>4 months of age), and are not permissive for hypertrophy in aged anabolic resistant mice (>24 months of age, refer to Lee et al. [41]). *CSA* cross-sectional area

With this investigation, our laboratory has now evaluated the hypertrophic response to overload in young, mature, and aged mice in both the presence and absence of satellite cells. Satellite cell-mediated myonuclear accretion is required for overload-induced muscle fiber hypertrophy in young growing mice, is not necessary for hypertrophy in mature mice [4, 24–26], and cannot drive hypertrophy in aged anabolic-resistant mice (>24 months of age) (Fig. 5) [41]. The effects of satellite cell-mediated myonuclear accretion during muscle fiber hypertrophy therefore exist along a dynamic continuum throughout the lifespan.

## Conclusions

Young mice (2–2.5 months of age) require satellite cell-mediated myonuclear accretion in order to undergo overload-induced hypertrophy. Furthermore, young mice respond differently to overload compared to mature (>4 month old) mice; young mice do not demonstrate an increase in eMyHC+ fibers in the presence of satellite cells and do not have increased fiber number regardless of satellite cells. Elevated fiber number with overload in mature mice may be at least partially due to muscle fiber splitting and/or altered muscle geometry. However, increased fiber number in mature mice does not disguise the ~20% average muscle fiber growth in SC+ and SC- mice after overload, further supporting the notion that satellite cells are not required for hypertrophy in fully grown adult mice. Future investigations will focus on compensatory mechanisms that enable mature mouse muscle to hypertrophy in the absence of satellite cell-mediated myonuclear accretion.

## Additional files

Additional file 1: Example of 5-bromo-2′-deoxyuridine (BrdU) staining after 14 days of traditional synergist ablation overload (removal of large portion of gastrocnemius and soleus). The overall BrdU findings can be found in a previous investigation from our laboratory [4], but the relationship of BrdU staining to central myonuclei was not reported. Panels **a-d** illustrate that BrdU+ (white arrow) and BrdU- (blue arrows) central myonuclei can be found after overload, suggesting that this process may be dependent on or independent from satellite cell proliferation. Pink arrows show a BrdU+ myonucleus that is not mispositioned. Staining protocols are found in McCarthy et al. [4]. Scale bar = 20 μm. (PDF 156 kb)

Additional file 2: Average muscle fiber cross sectional area (CSA) is shifted rightward with a consistent distribution in young and mature satellite cell-replete (SC+) and mature cell-depleted mice (SC-), but not young SC- mice after synergist ablation overload of the plantaris for 10 days (OV). Average muscle fiber CSA in **a** young SC+ sham (*n* = 7) and OV (*n* = 8) mice, **b** mature SC+ sham (*n* = 9) and OV (*n* = 6) mice, **c** young SC- sham (*n* = 6) and OV (*n* = 6) mice, **d** and mature SC- sham (*n* = 7) and OV (*n* = 7) mice. (PDF 129 kb)

Additional file 3: Hypertrophic response after synergist ablation overload of the plantaris for 10 days (OV) in young vehicle- and tamoxifen-treated Pax7-CreER mice (*n* = 2 F/1 M per group, with the appropriate fiber size correction applied to females [26]). Tamoxifen treatment does not affect **a** muscle fiber size or **b** satellite cell density after overload. (PDF 30 kb)

Additional file 4: Body weight (**a-b**), absolute plantaris muscle wet weight (**c-d**), and plantaris wet weight normalized to body weight (**e-f**) in young and mature satellite cell-replete (SC+) and -depleted (SC-) mice after synergist ablation overload of the plantaris for 10 days (OV). (PDF 41 kb)

Additional file 5: Muscle fiber splitting after 14 days of mechanical overload. Panels **a-d** illustrate fiber splitting over ~50 μm span on serial cross sections of a frozen plantaris muscle. Representative images visualize laminin (red) and dystrophin (green) to identify muscle fiber borders, and myonuclei (blue). Blue arrows point to central myonuclei that manifest prior to the appearance of each new branch in the muscle fiber (white arrows). The inset in panel **c** shows a partial fiber border (laminin+/dystrophin+) that arises during the splitting process on cross-sections. Panel **e** shows a phase-contrast image of a trifurcated single muscle fiber from this same mouse, along with myonuclei (blue). Panel **f** illustrates the extent of fiber splitting morphology in this mouse, with minimal eMyHC expression (pink muscle fiber, denoted with pink arrow). Note that many fibers contain >1 mispositioned myonucleus. Immunohistochemistry images were captured at ×20 and ×40 magnification, and single fiber image was captured at ×40; Scale bars = 50 μm. (PDF 3413 kb)

## Abbreviations

CN: Central nucleus
CSA: Cross-sectional area
DAPI: 4′,6-Diaminidino-2-phenylindole
DTA: Diphtheria toxin A
ECM: Extracellular matrix
eMyHC: Embryonic myosin heavy chain
HRP: Horseradish peroxidase
IgG: Immunoglobulin G
IHC: Immunohistochemistry
MoM: Mouse-on-Mouse
OV: Overload
Pax7: Paired box 7
PBS: Phosphate-buffered saline
TSA: Tyramide signal amplification
SC: Satellite cell
Tam: Tamoxifen
Veh: Vehicle
