# Supplementary figures and images for: Differential requirement for satellite cells during overload-induced muscle hypertrophy in growing versus mature mice
Source: Skelet Muscle. 2017 Jul 10;7:14. doi: 10.1186/s13395-017-0132-z (PMC5504676; doi:10.1186/s13395-017-0132-z)

**Dystrophin** **DAPI** **BrdU**  
**14 day OV**

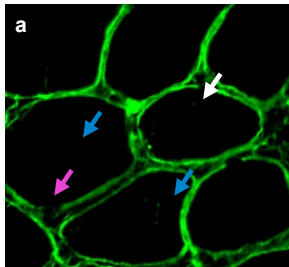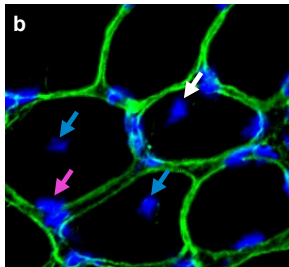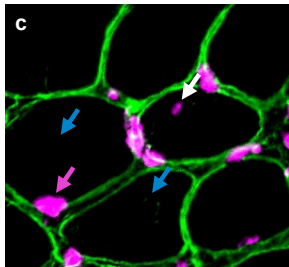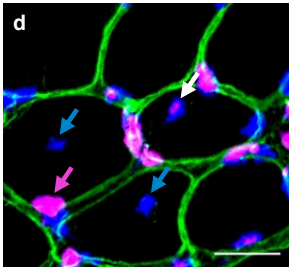

Supplement: Supplementary file 1 — Example of 5-bromo-2′-deoxyuridine (BrdU) staining after 14 days of traditional synergist ablation overload (removal of large portion of gastrocnemius and soleus). The overall BrdU findings can be found in a previous investigation from our laboratory [4], but the relationship of BrdU staining to central myonuclei was not reported. Panels a-d illustrate that BrdU+ (white arrow) and BrdU- (blue arrows) central myonuclei can be found after overload, suggesting that this process may be dependent on or independent from satellite cell proliferation. Pink arrows show a BrdU+ myonucleus that is not mispositioned. Staining protocols are found in McCarthy et al. [4]. Scale bar = 20 μm. (PDF 156 kb) [file 13395_2017_132_MOESM1_ESM.pdf]

**a**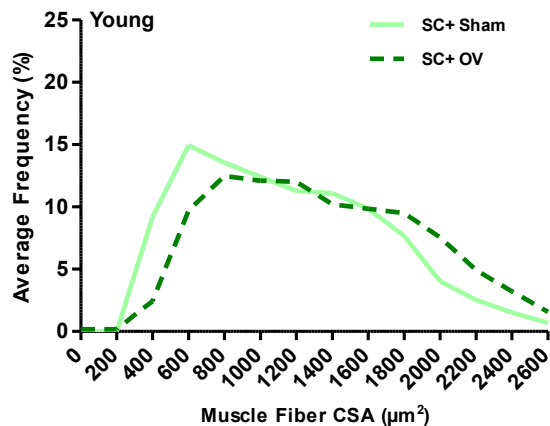**b**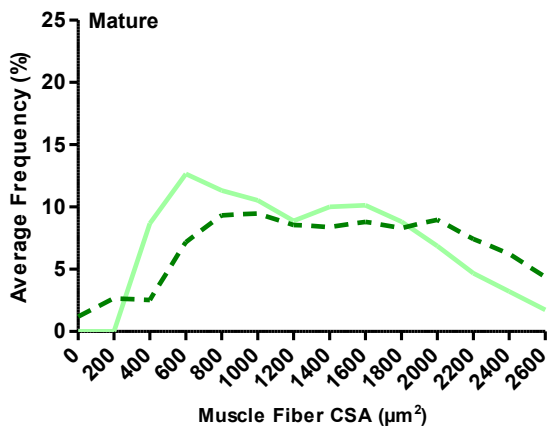**c**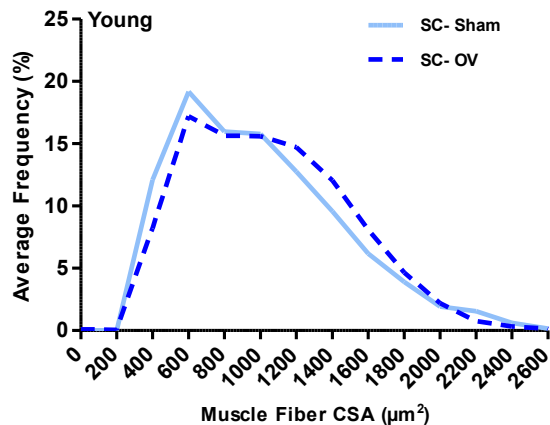**d**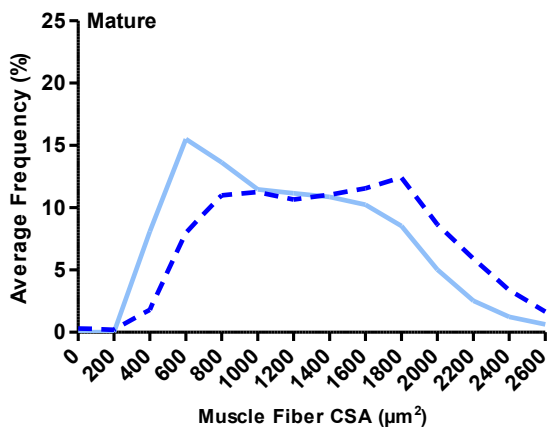

Supplement: Supplementary file 2 — Average muscle fiber cross sectional area (CSA) is shifted rightward with a consistent distribution in young and mature satellite cell-replete (SC+) and mature cell-depleted mice (SC-), but not young SC- mice after synergist ablation overload of the plantaris for 10 days (OV). Average muscle fiber CSA in a young SC+ sham (n = 7) and OV (n = 8) mice, b mature SC+ sham (n = 9) and OV (n = 6) mice, c young SC- sham (n = 6) and OV (n = 6) mice, d and mature SC- sham (n = 7) and OV (n = 7) mice. (PDF 129 kb) [file 13395_2017_132_MOESM2_ESM.pdf]

**a**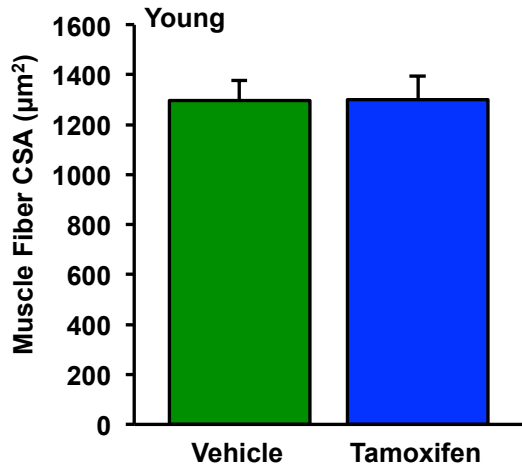**b**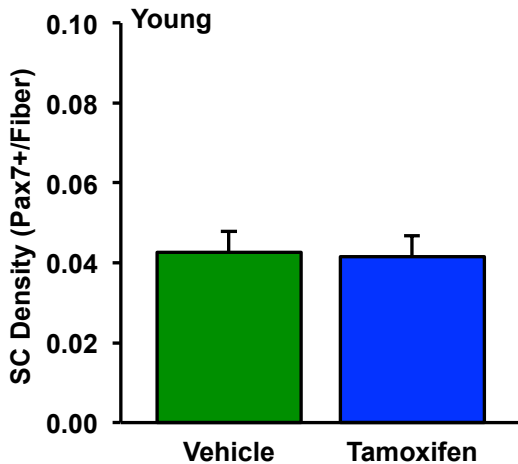

Supplement: Supplementary file 3 — Hypertrophic response after synergist ablation overload of the plantaris for 10 days (OV) in young vehicle- and tamoxifen-treated Pax7-CreER mice (n = 2 F/1 M per group, with the appropriate fiber size correction applied to females [26]). Tamoxifen treatment does not affect a muscle fiber size or b satellite cell density after overload. (PDF 30 kb) [file 13395_2017_132_MOESM3_ESM.pdf]

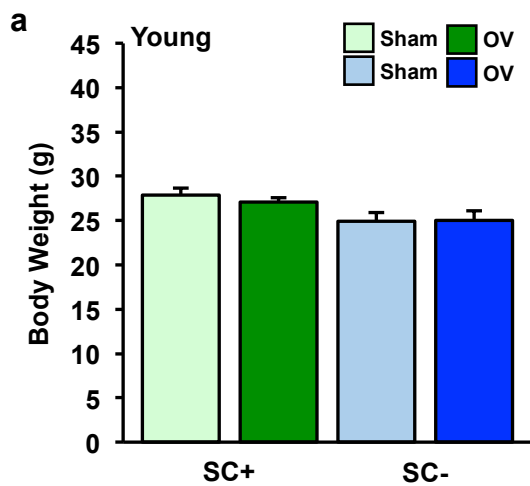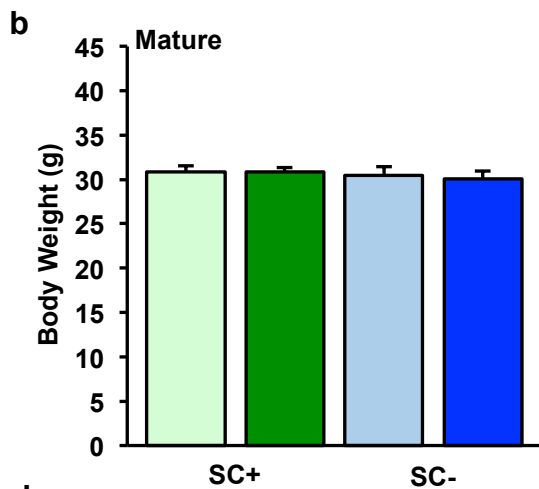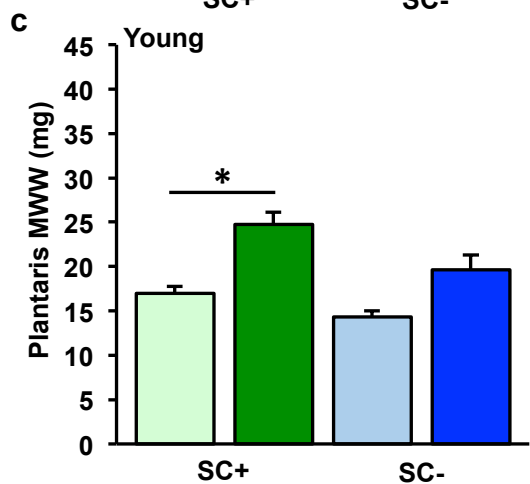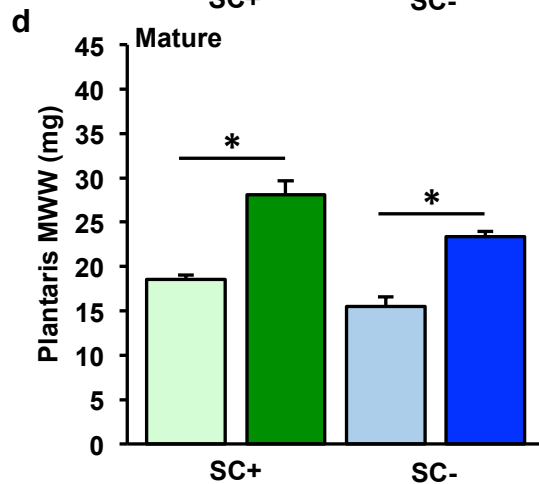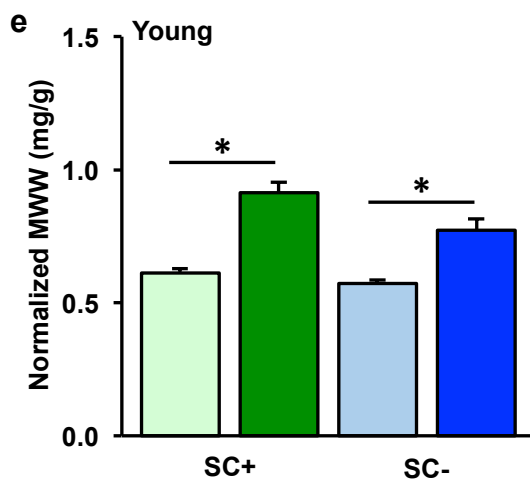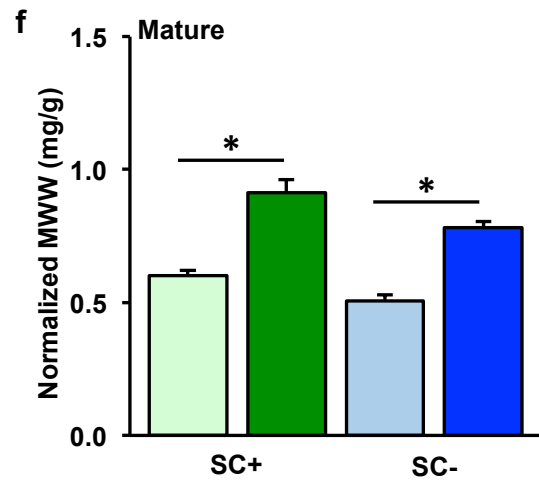

Supplement: Supplementary file 4 — Body weight (a-b), absolute plantaris muscle wet weight (c-d), and plantaris wet weight normalized to body weight (e-f) in young and mature satellite cell-replete (SC+) and -depleted (SC-) mice after synergist ablation overload of the plantaris for 10 days (OV). (PDF 41 kb) [file 13395_2017_132_MOESM4_ESM.pdf]

Laminin Dystrophin DAPI

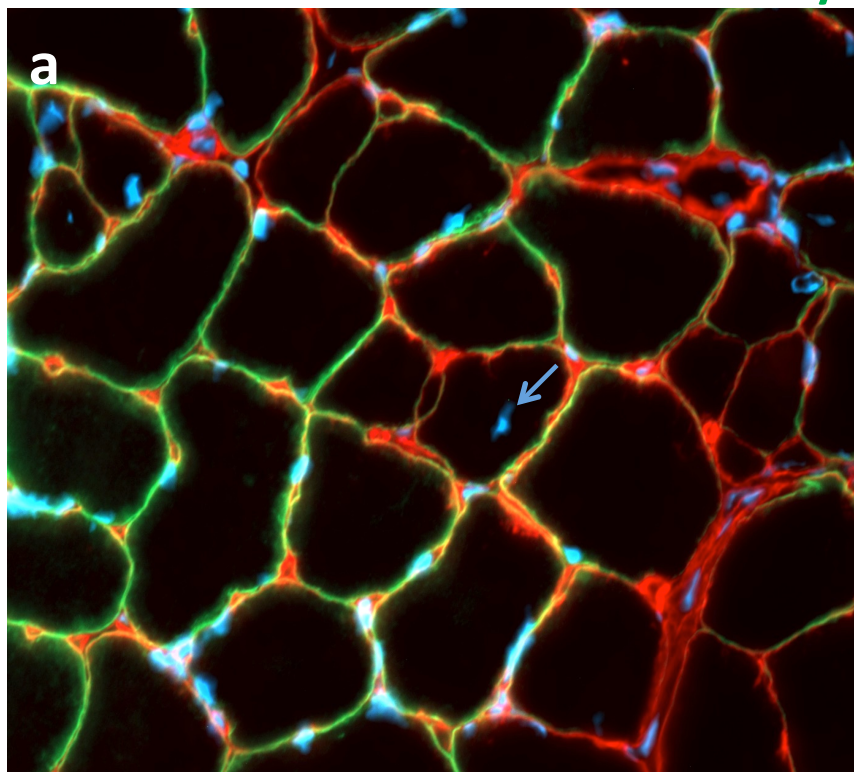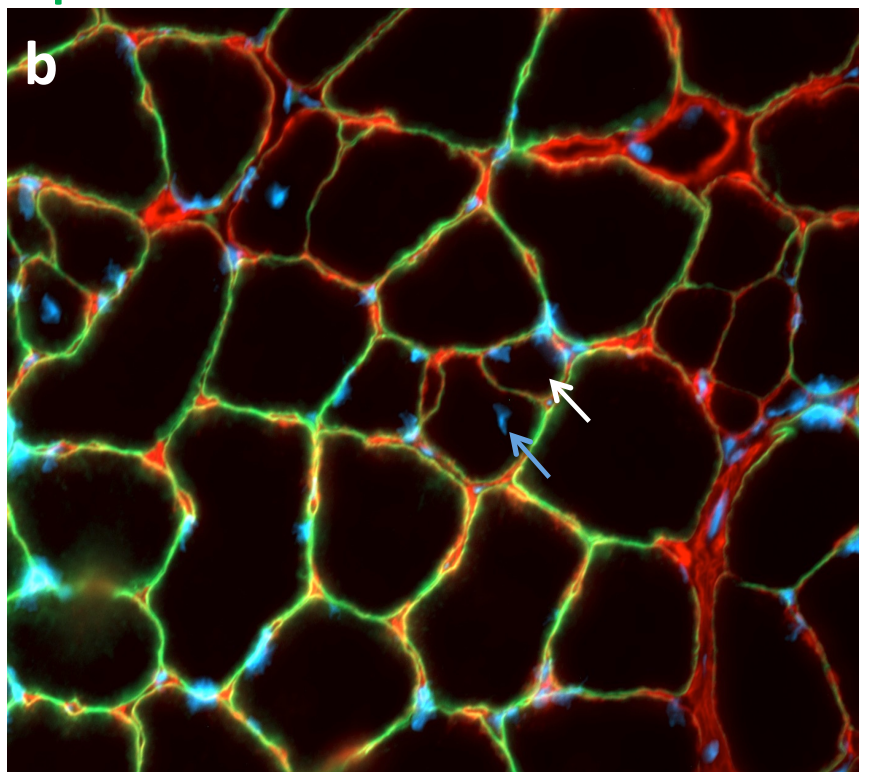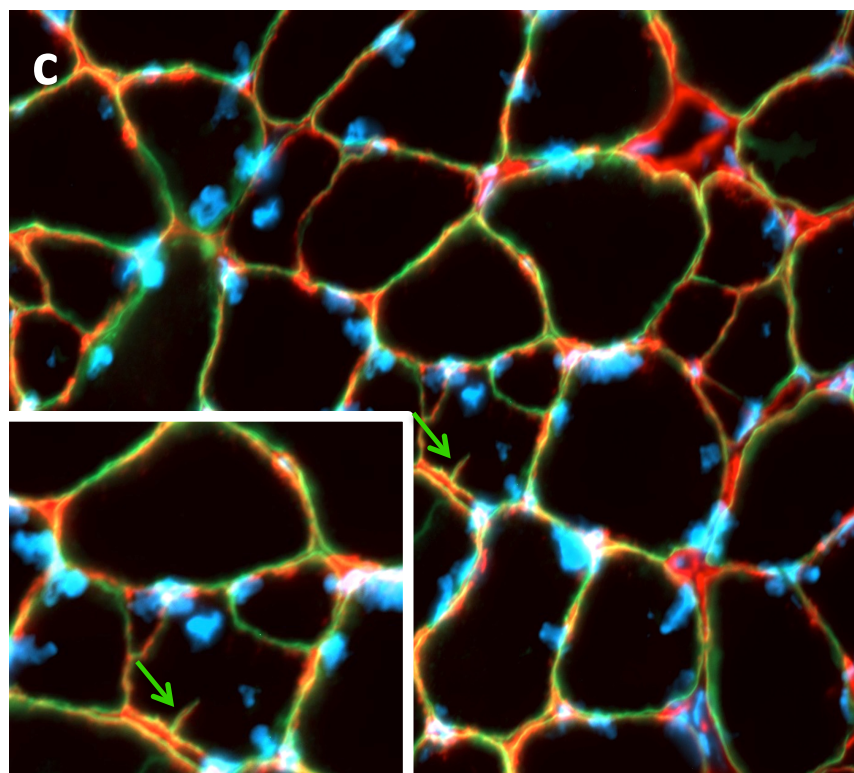

DAPI

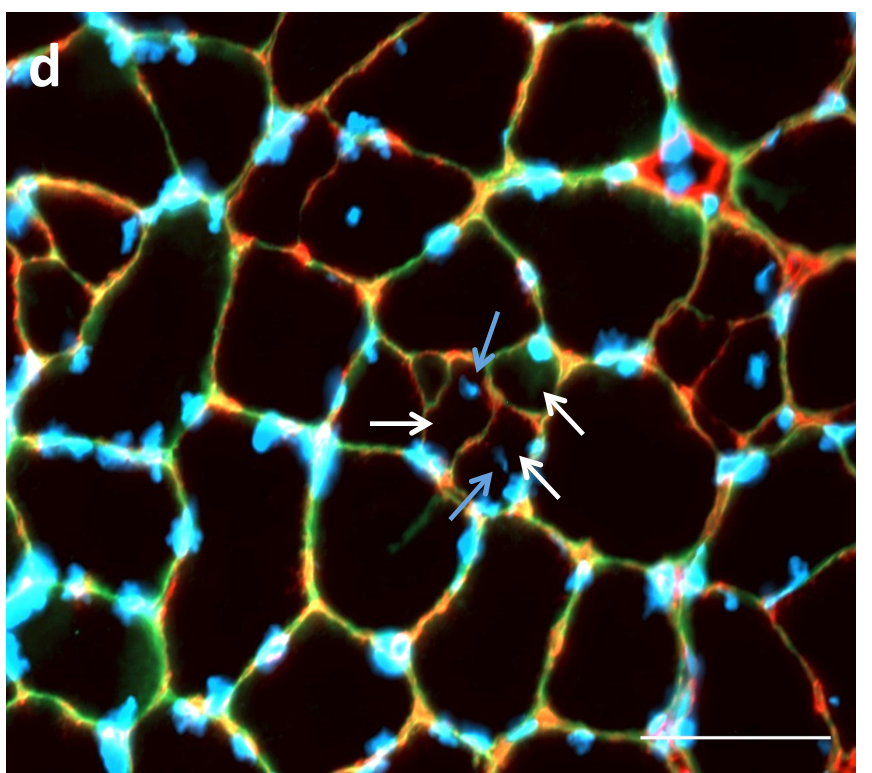

Laminin DAPI eMyHC

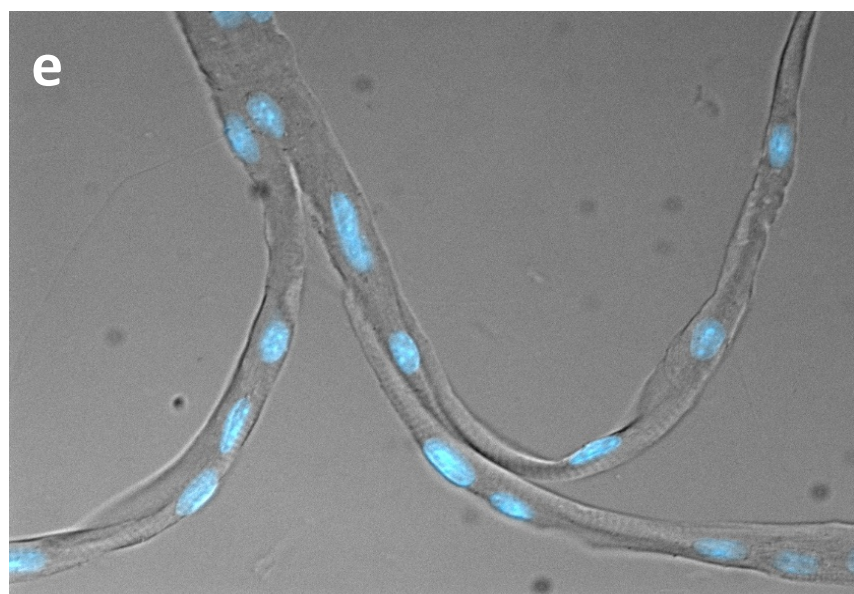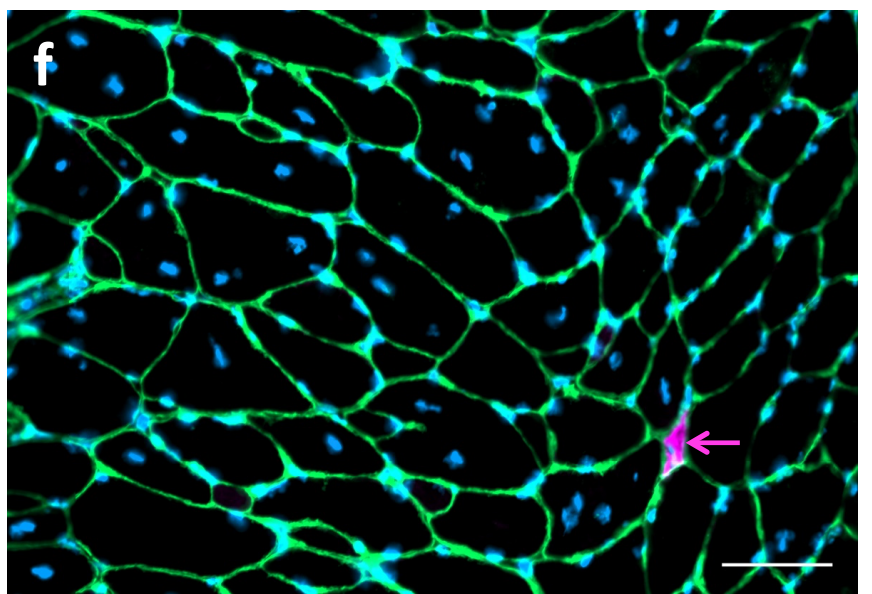

Supplement: Supplementary file 5 — Muscle fiber splitting after 14 days of mechanical overload. Panels a-d illustrate fiber splitting over ~50 μm span on serial cross sections of a frozen plantaris muscle. Representative images visualize laminin (red) and dystrophin (green) to identify muscle fiber borders, and myonuclei (blue). Blue arrows point to central myonuclei that manifest prior to the appearance of each new branch in the muscle fiber (white arrows). The inset in panel c shows a partial fiber border (laminin+/dystrophin+) that arises during the splitting process on cross-sections. Panel e shows a phase-contrast image of a trifurcated single muscle fiber from this same mouse, along with myonuclei (blue). Panel f illustrates the extent of fiber splitting morphology in this mouse, with minimal eMyHC expression (pink muscle fiber, denoted with pink arrow). Note that many fibers contain >1 mispositioned myonucleus. Immunohistochemistry images were captured at ×20 and ×40 magnification, and single fiber image was captured at ×40; Scale bars = 50 μm. (PDF 3413 kb) [file 13395_2017_132_MOESM5_ESM.pdf]
